# Transcatheter Embolization of Systemic-to-Pulmonary Collaterals: A New Approach Using Concerto™ Helix Nylon-Fibered Microcoils

**DOI:** 10.3390/jcm14010113

**Published:** 2024-12-28

**Authors:** Jochen Pfeifer, Martin Poryo, Anas Gheibeh, Axel Rentzsch, Hashim Abdul-Khaliq

**Affiliations:** Department of Pediatric Cardiology, Saarland University Medical Center, D-66421 Homburg, Germany

**Keywords:** systemic-to-pulmonary collaterals, major aorto–pulmonary collateral arteries, embolization, coil, microcoil, congenital heart disease, single ventricle, Fontan circulation

## Abstract

**Background:** Systemic-to-pulmonary collaterals (SPCs) are common in congenital heart disease (CHD). Particularly in single ventricle anatomy and Fontan circulation, SPC can both complicate the postoperative course and lead to clinical deterioration in the long term. The treatment of SPC is controversial. The aim of our study was (1) to retrospectively analyse patients who underwent SPC embolization using Concerto™ Helix nylon-fibred microcoils (CHMs) and (2) to describe the interventional technique. **Methods:** In this single-centre retrospective observational cohort study, we analysed clinical and imaging data of all patients who underwent transcatheter embolization of SPCs using CHMs from January 2016 to December 2023. **Results:** In 38 consecutive patients (65.8% male, median age 41 months, range 2–490), a total number of 141 CHMs had been implanted into 64 SPCs in 49 procedures. The majority were arterial SPCs (*n* = 59/64) originating from the thoracic aorta or its branches; 5/64 were veno-venous SPCs. Primary closure succeeded in all procedures. The CHM diameters ranged from 3 to 8 mm, with 5 mm being the most commonly used diameter. The mean coil/SPC ratio was 2.6 (range 1.3–5.3). CHM implantation was performed via four French sheaths. Both detachment and stable positioning were simple and safe. Neither non-target embolization nor coil migration occurred. One complication was a vascular injury with resulting extravasation of contrast medium. In 18/49 procedures (36.7%), coils other than CHMs or vascular plugs were additionally inserted into separate SPCs. **Conclusions:** CHMs are appropriate for SPC embolization in all age groups, including infants, with a low complication rate. The coils are particularly suitable for the closure of collaterals with a small diameter or tortuous course. They can be used in combination with other embolization devices to achieve comprehensive collateral closure.

## 1. Introduction

Systemic-to-pulmonary collaterals (SPCs) are frequently encountered in congenital heart disease (CHD). Also referred to as “major aorto–pulmonary collateral arteries (MAPCA)”, arterial SPCs usually originate as irregular vessels from the thoracic aorta or its branches, namely, from the subclavian, internal mammary, thyroid, or carotid arteries [1]. Venous SPCs often derive from the caval or other main thoracic veins. Typically, SPCs occur in cyanotic CHD with impaired pulmonary perfusion, especially in univentricular physiology and cavo–pulmonary anastomoses. Chronic hypoxaemia as well as reduced or non-pulsatile pulmonary blood flow and inflammatory effects are assumed to promote the development of SPC [2,3]. Moreover, premature neonates and infants can be affected by arterial collaterals. In noncyanotic CHD, SPCs are rare [4]. Nevertheless, the pathogenetic factors are still not completely understood.

Irregular collaterals emerge in a great variety of shapes, sizes, and numbers. They may present as unifocal or multifocal singular vessels as well as highly branched collateral networks. Due to the markedly different individual characteristics, a general classification of SPCs is difficult [5,6].

In the case of collateral-dependent pulmonary perfusion such as in selected types of pulmonary atresia, arterial SPCs are already developing during the embryonic phase and are considered to be essential SPCs. Non-essential SPCs may evolve at any stage in complex CHD from infancy to adulthood. The latter collaterals are responsible for excessive pulmonary blood flow resulting in hyperaemia and increased pulmonary pressure with the risk for haemoptysis or severe haemorrhages [7]. As well, cardiac volume and pressure overload may occur [8].

The treatment of non-essential arterial and venous SPCs is handled very differently [2]. In many centres, SPCs are the target of consistent percutaneous embolization. For transcatheter occlusion, different devices are used such as coils, vascular plugs, duct and septal occluders, or particles [2,8,9,10]. Selecting the appropriate method of SPC embolization is a challenge that depends on the location, size, and course of the collaterals as well as the availability of devices suitable in size. Individual solutions have to be chosen. SPCs with small diameters that can nevertheless feed extensive collateral networks as well as tortuous SPCs may particularly be difficult to embolize. In these cases, Concerto™ Helix nylon-fibred microcoils (CHM) are used in our institution, which, to our knowledge, has not yet been reported.

The aim of our study was to describe the technique and to evaluate the applicability, efficacy, safety, and potential complications of using CHMs for the embolization of SPCs. The patient population as well as the sizes and numbers of the implanted coils, primary outcome, and any complications of the embolization were analysed.

## 2. Materials and Methods

### 2.1. Study Design

This retrospective single-centre study was performed at a tertiary care medical centre (Saarland University Medical Center, Homburg, Germany), after approval from the local ethics committee of the Saarland, Saarbrücken, Germany (file number 154/23). The authors declare no conflicts of interest. There are neither contracts with companies nor are funds provided by the industry.

**Figure 1:**
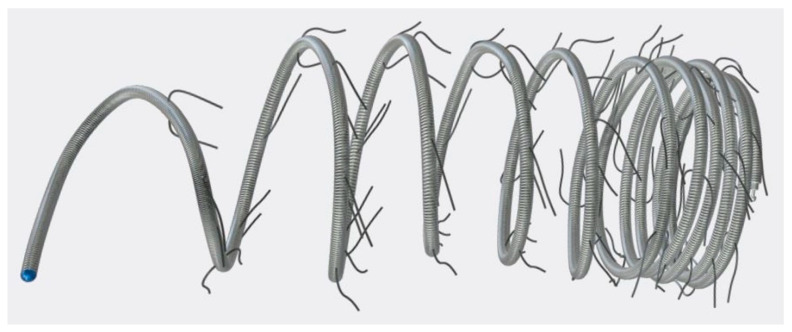
Concerto™ Helix nylon-fibered microcoil (source: Concerto Coils Brochure, medtronic.com, with kind permission).

We included all patients who underwent transcatheter embolization of SPCs using Concerto™ Helix nylon-fibered microcoils (CHM; Micro Therapeutics, Inc., ev3 Neurovascular, Irvine, CA, USA) in our institution from January 2016 to December 2023. The mechanically detachable CHMs consist of a platinum alloy and are provided with nylon fibres. They are available in various diameters (from 2 to 10 mm) and lengths (from 3 to 30 cm). Figure 1 shows a CHM.

Parents or patients of legal age had given written informed consent for the intervention. Based on the retrospective nature of this data analysis, written informed consent for the study was waived.

We reviewed the relevant patients’ files as well as the angiographic records and post-interventional x-rays in order to obtain the following data:

- Patients’ characteristics (sex, body weight, and age at intervention);
- Ventricular morphology (biventricular or univentricular anatomy);
- Preceding surgery;
- Origin and diameter of the embolized SPCs;
- Number and size of the implanted CHMs;
- Oversizing of the CHMs;
- Primary success of the embolization;
- Procedure time (defined as time from the insertion of the sheath to the removal of the sheath) and fluoroscopy time;
- Contrast dose and radiation exposure (dose area product);
- Follow-up;
- Complications (vascular injuries, haemorrhage, coil migration, non-target embolization, thrombosis, embolism, and mortality);
- Use of plugs or coils other than CHMs for SPCs.

### 2.2. Catheterization and Transcatheter Embolization

Under sedation, percutaneous catheterization was performed via femoral vessel puncture and the insertion of 4 French sheaths. Four French angiographic catheters (Multipurpose A Special or MPA 2 or Pigtail; Cordis Corporation, Miami Lakes, FL, USA) were used for the angiography of the thoracic aorta and its branches, respectively, the caval and the brachiocephalical veins, as well as the SPCs in order to locate the collaterals. The contrast agent (Solutrast™ 300, Bracco Imaging, Konstanz, Germany) was selectively injected by hand into the collaterals to obtain a detailed depiction of the morphology and course of the SPCs and to measure their diameters. An angiogram of the pulmonary arteries was also performed. The indication for SPC closure was provided if they supplied pulmonary parenchyma, if pulmonary vascular contrast was visible after selective SPC angiography, or if there was an antegrade perfusion washout in the pulmonary arterial angiography (in accordance with previously described studies). The embolizations were performed regardless of the oxygen saturation values or pulmonary pressure values. To achieve a probing of the SPC as deep as possible, guide wires (0.014″, Whisper™ ES or LS; Abbott Medical, Santa Clara, CA, USA) and microcatheters (Rebar™-18; Micro Therapeutics, Inc., ev3 Neurovascular, Irvine, CA, USA) were used. Through the latter, the CHMs were pushed and deployed into the target vessel aiming at a densely packed coil formation. They were then mechanically released by retracting the restraining wire within the pusher. The correct position and the required configuration of the CHM were fluoroscopically assessed during the intervention. Following this, the residual contrast medium flow and potential extravasation were controlled by selective SPC angiography to review the effectiveness as well as potential vessel injury. Successful closure was defined as total or subtotal occlusion (i.e., minimal residual contrast shunt). Heparin (100 IU per kilogram body weight) and antibiotic prophylaxis were administered during the procedure. Post-intervention, the patients were monitored in hospital for 48 h before discharge. A sports ban was imposed for 4 weeks depending on age. Clinical follow-ups and echocardiographies were carried out 4 weeks and 3 months after intervention, thereafter in individual intervals.

Possible coil migration was evaluated by follow-up chest X-ray or in subsequent angiographies.

CHMs have been preferred over other devices (e.g., different coils or vascular plugs) in SPCs with small diameters (≤4 mm) as well as in those with tortuous morphology or if probing was only possible using microcatheters. The lengths of the landing zones are usually difficult to measure in tortuous and curved SPCs. Therefore, they were mostly estimated in relation to the diameter. Multiple CHMs were inserted if the landing zones were not completely filled by the first one or to occlude additional collateral branches. The CHM has to be oversized in order to ensure a stable coil position within the target. The minimum oversize recommended by the manufacturer for all coil sizes is 1.43 times.

The fluoroscopy system used was Artis Q™ biplane (Siemens Healthineers, Forchheim, Germany).

### 2.3. Statistics

IBM© SPSS 28.0 statistics software and Microsoft© Excel 2016 were used for the statistical analyses. The oxygen saturation pre- and post-intervention was evaluated using paired *t*-tests. The normality of the data was assessed by a Shapiro–Wilk test.

Data analysis is purely descriptive. The data are given as an absolute number and percentage or median and range.

## 3. Results

### 3.1. Patients’ Characteristics, Contrast Doses, and Radiation Exposure

In 38 consecutive patients (thereof 65.8% male), CHMs had been implanted into 64 SPCs in a total of 49 procedures. In 29 patients, only one procedure was performed. Two separate sessions were performed for seven patients, and three sessions for two patients, each at different ages. The median age at the time of intervention was 41 months (range 2–490), and the median body weight was 13.2 kg (range 4.2–64.0). Multiple interventions may have been required in the same patient, as new SPCs can develop over time, which is particularly common in cyanotic CHD and Fontan circulation. In these cases, angiographic evaluation is necessary at different stages of surgical palliation. The cases are described in detail in Table 1.

**Figure 2:**
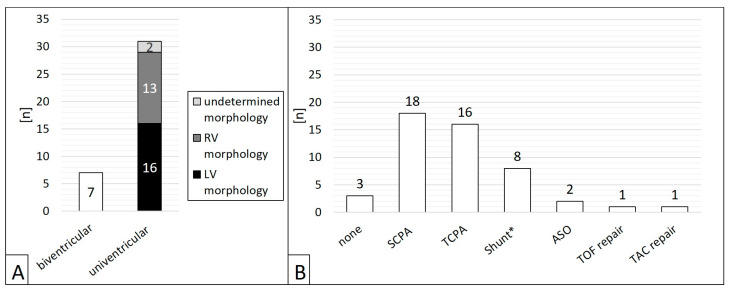
Patients’ ventricular morphology (**A**) and operations preceding the interventions (**B**). ASO = atrial switch operation; LV = left ventricle; SCPA = partial cavo–pulmonary anastomosis; RV = right ventricle; TAC = truncus arteriosus communis; TCPA = total cavo–pulmonary anastomosis; TOF = tetralogy of Fallot; * Blalock-Taussig shunt or central aorto–pulmonary shunt.

Of 38 patients, *n* = 31 (81.6%) had CHD with single ventricle anatomy (Figure 2A). Regarding the procedures, a single ventricle heart was present in 81.6% (*n* = 40/49). 46 interventions (93.9 %) were preceded by cardiac surgery (Figure 2B), in most cases (*n* = 34/49, 69.4%) a superior cavo-pulmonary anastomosis (SCPA) or a total cavo-pulmonary anastomosis (TCPA, Fontan circulation).

All procedures were performed electively. In patients with complex cardiac defects, the catheterizations were performed either for routine hemodynamic assessment before surgery or for follow-up after TCPA, arterial switch operation, or the repair of a tetralogy of Fallot or a common arterial trunk. In one case, relevant SPCs were found during catheterization to close a patent arterial duct. There were neither emergency interventions nor cases of previous haemoptysis.

The procedure time had a mean of 90.1 (± 26.1, median 85) minutes and the fluoroscopy time had a mean of 22.4 (± 8.5, median 20.5) min. The average contrast dose was 4.6 (± 3.5, median 3.7) mL/kg body weight. In terms of radiation exposure, the dose area product had a mean of 1151.6 (± 1795.2, median 413.6) cGy·cm^2^.

### 3.2. Characteristics of the Target Vessels, Characteristics of the Used Coils, and Coil Oversizing

**Figure 3:**
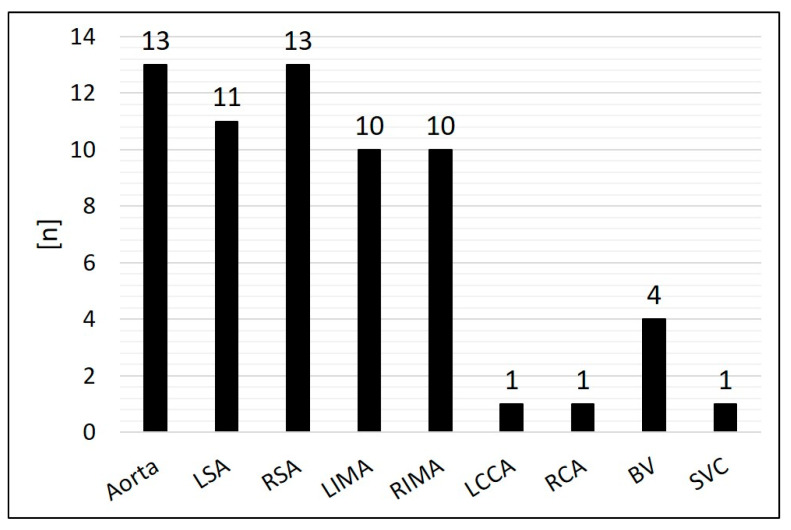
Distribution of the originating vessels of the systemic-to-pulmonary collaterals (SPCs). BV = brachiocephalic vein; LCCA = left common carotid artery; LSA = left subclavian artery; LIMA = left internal mammary artery; RCA = right coronary artery; RSA = right subclavian artery; RIMA = right internal mammary artery; SVC = superior vena cava.

**Figure 4:**
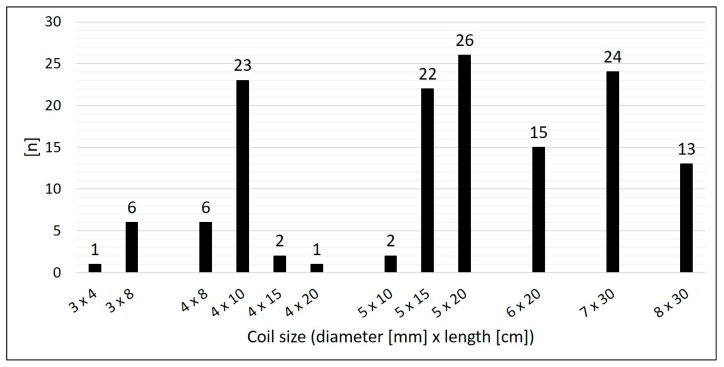
Size distribution of the used coils.

Arterial SPCs originating from the thoracic aorta or its branches were present in 92.2% (*n* = 59/64); 7.8% (*n* = 5/64) were venous SPCs from the superior vena cava or from the brachiocephalic vein (Figure 3). The median diameter of the target SPC was 1.95 mm (range 1.0–3.7). A total number of 141 CHM were inserted. The coil diameters were 3 to 8 mm, with 5 mm (*n* = 50; 35.5%) being the most commonly used; 3 mm was used in *n* = 7 (5.0%), 4 mm in *n* = 32 (22.7%), 6 mm in *n* = 15 (10.6%), 7 mm in *n* = 24 (17.0%), and 8 mm in *n* = 13 (9.2%) (Figure 4).

The median oversize of the coil diameter compared to the collateral diameter (CHM/SPC ratio) was 2.6-fold (range 1.3–5.3).

### 3.3. Use of Devices Other than CHMs

In 18 out of the 49 procedures, coils other than the CHM type or vascular plugs were additionally inserted into separate SPCs.

For short landing zones and when the SPC could not be probed deeply, *n* = 28 MReye™ Flipper™ detachable coils (Cook™ Medical, Bjaeverskov, Denmark) were used with diameters of 3 or 5 mm to prevent the longer CHM from protruding into the feeding vessel. A total of *n* = 7 Amplatzer™ Vascular Plugs (Abbott Medical, Plymouth, MN, USA) were used for SPCs with diameters larger than 4 mm and in long, non-tortuous landing zones.

### 3.4. Outcome, Complications, and Follow-Up

**Figure 5:**
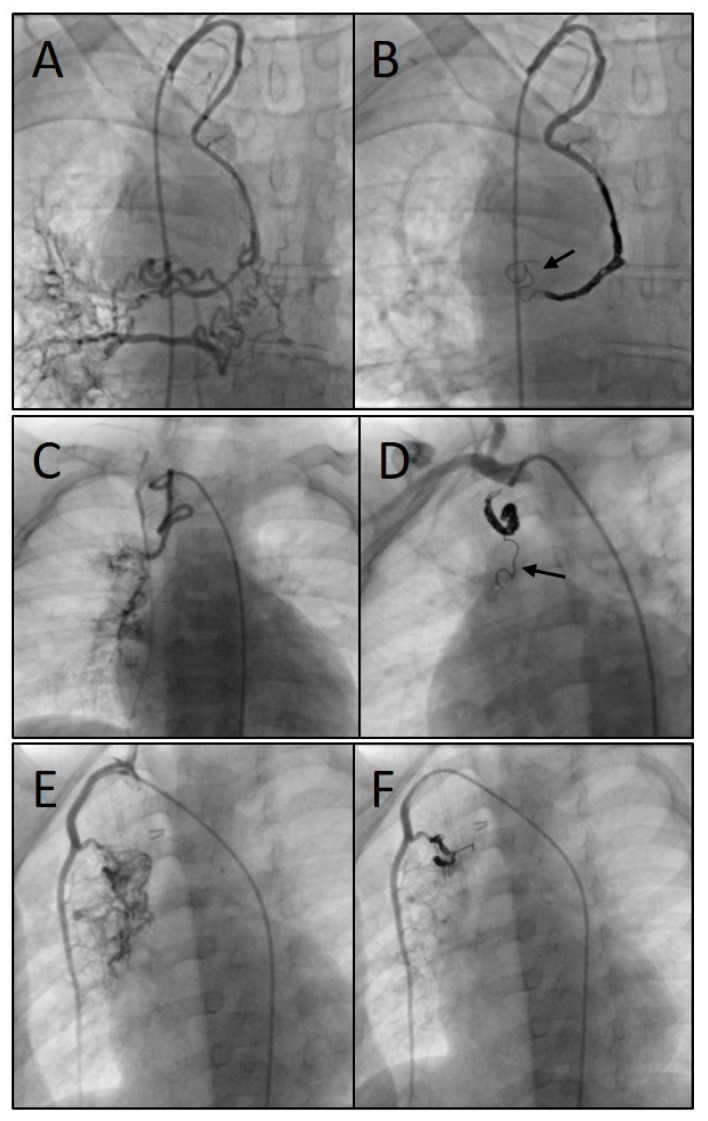
Angiograms of three different procedures showing the embolization of arterial systemic-to-pulmonary collaterals (SPCs): SPCs originating from the right subclavian artery (**A**), embolized with three coils (7, 8, and 8 mm) (**B**); SPCs originating from the right subclavian artery (**C**), embolized with three coils (4, 4, and 5 mm) (**D**); and SPCs from the right internal mammary artery (**E**), embolized with two coils (both 5 mm) (**F**). Arrows indicate the stretched configuration of the distal coil portion.

**Figure 6:**
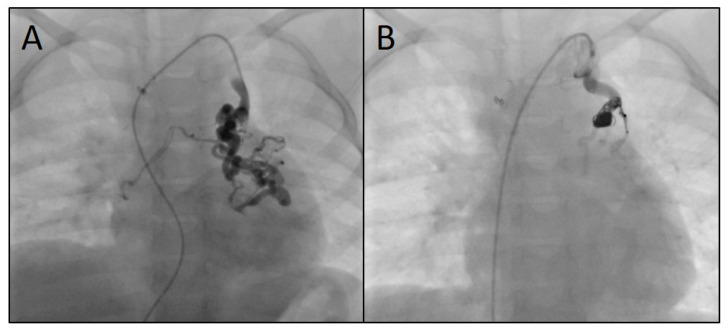
Angiograms of the embolization of a venous systemic-to-pulmonary collateral originating from the left brachiocephalic vein (**A**) using three microcoils (4, 7, and 8 mm) (**B**).

**Figure 7:**
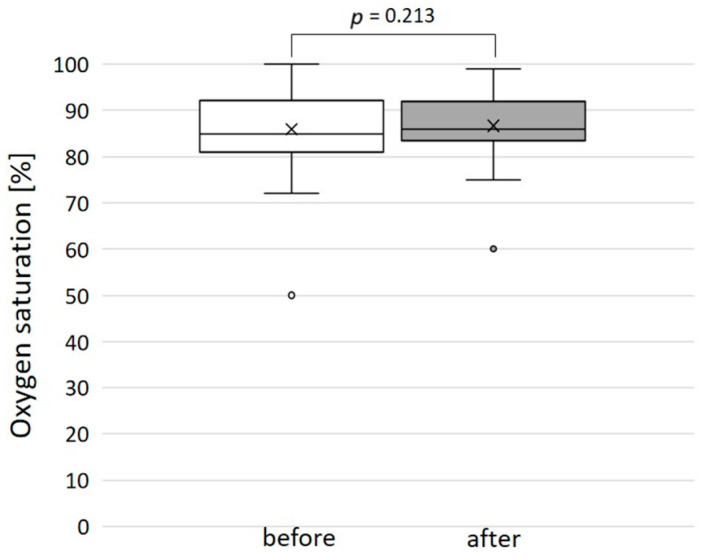
Oxygen saturation before and after the embolization of the systemic-to-pulmonary collateral: no significant change.

The immediate technical success rate was 100% with total or subtotal closure of the SPCs in all patients. Figure 5 shows three angiograms illustrating embolizations of arterial SPCs; Figure 6 shows a venous SPC embolization. In 24/49 (49.0%) procedures, a small portion of the distal CHMs formed a stretched instead of a coiled structure (see Figure 5B,D). However, the remaining proximal part of the CHMs could be stably deployed even in these cases and formed the desired densely packed configuration. In 47 of 49 cases, follow-up chest X-ray or fluoroscopy were performed where a persistently stable CHM position could be detected in all cases. Neither non-target embolization nor coil migration occurred during the intervention or in the follow-up. Notably, the mean oxygen saturation, which was 86% before the intervention and 87% after the intervention, did not change significantly (Figure 7).

Thrombosis or embolism did not occur. There was no mortality. Post-procedural pulse loss did not occur in the patients. The duration of hospital stays (discharge after 48 h of monitoring) was not longer than usual in our institution for patients undergoing interventional catheterization.

**Figure 8:**
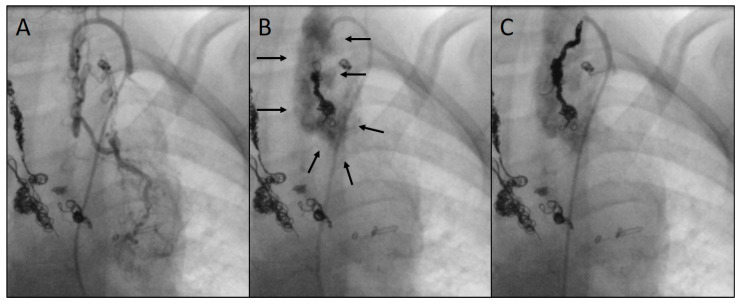
Angiograms of an embolization complicated by a vascular injury: depiction of an arterial systemic-to-pulmonary collateral (SPC) from the left subclavian artery (**A**); perivascular extravasation (indicated by arrows) following the insertion of the first coil (5 mm) (**B**); implantation of two more coils (6 and 7 mm) into the residual landing zone with complete closure of the SPC and cessation of extravasation (**C**). Previously implanted coils can be seen on the left side of the images.

As a complication, there was one vascular injury in a patient who underwent CHM embolization of an SPC originating from the left subclavian artery (Table 1, procedure no. 10). After deploying the first CHM (diameter 5 mm, oversize 3.1-fold), there was a contrast extravasation surrounding the target vessel. Two more CHMs were promptly inserted into the proximal landing zone, whereupon no more extravasation was detectable (Figure 8). The patient was stable during the entire procedure as well as in the post-interventional monitoring. Arterial hypotension, tachycardia, a decrease in haemoglobin level, pain, or other clinical symptoms did not occur. The coils remained stable in the desired position.

For all patients, median follow-up time was 63.0 months (range 0.5–96.0). During this time, no complications related to the SPC embolization such as late coil migration occurred. Independent of the procedure, a child with a tetralogy of Fallot and borderline left ventricle died three weeks later after surgical correction and postoperative extracorporeal life support (day 20 postoperative).

## 4. Discussion

Abnormal blood supply to the lungs in patients with severe congenital heart or vascular malformations leads to pulmonary hyperaemia and increased pulmonary resistance particularly when undergoing several stages of surgical palliation. Moreover, systemic-to-pulmonary collaterals (SPCs) lead to increased pulmonary venous returns during cardiac surgery and cardiopulmonary bypass. Several approaches are used to close abnormal vessels during interventional catheterizations.

We report on our institutional experiences with a novel approach to the transcatheter embolization of SPCc using Concerto™ Helix nylon-fibered microcoils (CHMs). With a total number of 141 CHMs, 64 SPCs had been occluded in 38 consecutive patients. Embolization was successful in all cases. The duration of hospital stay was not prolonged compared to other interventional SPC embolization procedures in our institution. The vast majority of the SPCs (*n* = 57/64) derived either from the aorta, the subclavian, or internal mammary arteries which are also the most common origins according to the published literature [1,11]. There was one complication in the form of a vascular injury with resulting contrast medium extravasation. When probing SPCs, it has to be taken into account that these are vulnerable vessels with abnormally thin vascular walls, and that vasoconstriction can occur during the intervention. Careful vessel probing should be performed using microcatheters aiming at distal coil implantation. Especially small SPCs, which nevertheless may feed extensive collateral networks, as well as highly tortuous SPCs can be embolized selectively using this technique. In spite of the above-mentioned complication, CHMs appear to be particularly suitable due to their soft, flexible, and adaptable structure. Severe complications did not occur in the described population. It is assumed that embolizations as distal as possible prevent the recurrence of SPC, which is achieved well using particle embolization according to previous studies [9]. In long landing zones with additional branches, several CHMs should be inserted if necessary. The embolization of the entire internal mammary arteries as feeding vessels can be avoided by selective SPC closure. Brown et al. have previously reported on the favourable use of micro-catheters in difficult target vessels [12].

The delivery mechanism of the CHM was easy to operate, and there were no complications associated with the coil release in the described population. CHMs are pre-assembled, mechanically detachable coils. After positioning, they are released by simply retracting the retaining wire. In comparison, there are numerous mechanisms for other types of coils. For example, the otherwise used Cook™ coils must first be screwed to the retaining wire. After insertion, they must be unscrewed to release them, which can be complicated with tortuous vessels. Another option is the use of pushable coils, which are more difficult to insert in a controlled manner. In contrast, detachable coils can be pulled back into the catheter before release if they are in an unfavourable position. Nevertheless, dislocation, non-target embolization, and coil migration are also possible complications with CHMs. In the case of arterial SPCs, there is a risk for systemic arterial embolism, for example in the extremities or in the brain, resulting in ischemia and infarction. In venous SPC embolization, the dislocation of released coils into pulmonary arterias via cavo–pulmonary connections may occur, which can be deleterious particularly in patients with Fontan circulation. Strict intra- and post-interventional clinical monitoring is necessary in order to recognise adverse events at an early stage. There must be the possibility of emergency interventional or surgical retrieval of migrated coils [13,14]. In the population described above, neither non-target embolization nor coil migration occurred. However, we have not carried out a comparison with other available detachable microcoils, so we cannot conclude whether CHMs are advantageous over them.

It appeared advantageous that the deployed CHMs are densely packed within the SPCs and thus adapt to the course of the SPCs forming an almost “cast-like” structure. As a result, extensive vessel obstruction occurs without delay. The initial portion of the CHM may be inserted in a stretched shape without forming a coiled configuration. A possible cause for this may be that the SPCs usually taper distally so that the coils cannot unfold. This does neither affect the stability nor the efficacy of the CHMs as long as the main part takes the desired shape. Advantageously, the nylon fibres have been shown to promote faster thrombin generation, which should lead to definitive SPC closure [15,16].

Both the tapering diameters of the collaterals and the potential vasoconstriction during the angiography make it difficult to select the appropriate CHM size. Therefore, the selection depended to a certain extent on the interventionalist’s experience. Coil oversizing results in stable positioning similar to other types of embolizing devices [10]. Given the 1.3 to 5.3-fold diameter of the CHMs versus the SPCs in our patients, a wide range of CHM/SPC ratios appears to be acceptable. The large selection of available CHM sizes has to be seen as an advantage especially because both the sizes and morphologies of SPCs are widespread. In long landing zones, either long or multiple CHMs have to be used. Implantation close to the branching or even protruding into the feeding vessel must be strongly avoided in order to prevent dislocation and non-target embolization. As the CHM can be inserted via small catheters and thus via small vascular accesses, they are not only appropriate for children and adults of all age groups and body sizes, but also particularly for neonates. However, in 37% of our cases, additional devices other than CHMs had to be used to achieve complete collateral closure. The choice of the appropriate coil or vascular plug depends on the configuration, the course and the landing zone of the target vessel as well as on the experience of the interventionalist.

The duration of the procedures in our population had a median of 85 min (mean 90.1) compared with a median of 116 min described by Yeh et al. [17] and 71 min described by Haas et al. [18], both large studies of interventional congenital cardiac catheterization. Sadiq et al. [19] report a mean procedure time of 55.12 min and a fluoroscopy time of 20.78 min for SPC embolization with Cook coils in patients with a tetralogy of Fallot (compared to a fluoroscopy time of 22.4 min described above). In general, the procedural parameters appear to be difficult to compare as different interventions are described. In addition, the above-mentioned catheterizations involved additional diagnostics for complex CHD, which increases both procedure and fluoroscopy time.

The dose of contrast agent administered (median 3.7 mL/kg) was comparable to published data, e.g., the median dose was 3.9 mL/kg in the study of Senthilnathan et al. [20]. Furthermore, the radiation exposure of mean 1151.6 cGy·cm^2^ (median 413.6) in our population was similar to previous studies in children or lower: Chida et al. report 2242.4 cGy·cm^2^ in interventional catheterizations [21], and Cevallos et al. report a range of 249 to 17,990 cGy·cm^2^ in different interventions [22].

According to the published literature, the management of SPCs is handled very differently by interventional cardiologists [2,23]. On the one hand, earlier studies suggested that SPCs have no significant pathophysiological relevance or that new ones developed quickly after SPC closure anyway [24,25]. Furthermore, the haemodynamic significance of individual SPCs is undoubtedly difficult to measure [26]. Some studies suggest that SPCs can be better assessed by magnetic resonance imaging than by cardiac catheterization [27,28].

On the other hand, recent studies have shown that SPCs are associated with a higher morbidity in children with CHD. Especially for patients with SCPA and TCPA, SPCs are deleterious and are therefore consistently embolized in many centres [9,11]. Dori et al. demonstrated a favourable decrease in pulmonary blood flow after the closure of SPCs in SCPA patients [29]. With underlying passive pulmonary perfusion in these patients, counter flow via arterial collaterals can lead to impaired pulmonary blood flow. Prolonged postoperative hospital stays and a higher incidence of pleural effusion are described [30]. In the long term, failing Fontan circulation can be promoted [31,32,33]. As veno-venous collaterals are associated with cyanosis and an increased risk for plastic bronchitis, they should also be embolized in these patients [34,35]. Moreover, SPCs are responsible for morbidity as well in other CHD, e.g., in children undergoing an arterial switch operation [36,37]. The majority of patients in the population described above had univentricular anatomy (*n* = 31/38), and most SPC embolizations were preceded by SCPA and TCPA surgery.

Of note, essential SPCs have to be strictly differentiated as they have to be conserved in collateral-depending pulmonary circulation and may have to be surgically unifocalized [38,39].

There are several limitations in the present study. The main limitations are the retrospective nature and the single-centre design of the study. The sample sizes of both the patients and the procedures are small. Although the SPC diameters were measured, the selection of the implanted coils depended also on the experience of the interventionalist. A specific recommendation regarding the CHM sizes is difficult to provide. Prospective studies with a larger population are required, particularly with regard to a comparison with other types of microcoils.

We conclude that Concerto™ Helix nylon-fibered microcoils are simple and safe to use with a high efficacy and a low complication rate. They are suitable in the embolization of systemic-to-pulmonary collaterals, particularly those with small diameters, highly tortuous morphologies, and variable landing zones. As they are inserted via small vessel accesses, they can be used in patients of a wide range of age groups and body weights. As an expansion of the arsenal, CHMs can be used in combination with other types of devices to achieve comprehensive collateral embolization in congenital heart defects.

## Figures and Tables

**Table 1:** Case-based description of diagnoses, ventricular morphology, preceding surgery, number and origin of the collaterals, coil characteristics, procedural duration, fluoroscopy time, contrast volume, dose area product, complications, and follow-up time.

Procedure No.	Sex	Age (Months)	Weight (kg)	Diagnosis	Univentricular Heart	Most Recent Surgery	Embolized SPC (*n*)	Origin of SPC	Diameter of SPC (mm)	Implanted Coils (*n*)	Size of Coil (Diameter (mm) × Length (cm))	Coil/SPC Ratio	Procedure Time (min)	Fluoroscopy Time (min)	Contrast Volume (mL)	Dose Area Product (cGy·cm^2^)	Complications	Follow-Up Time (Months)
1	M	11	9.0	TOF	No	TOF repair	1	Ao	1.4	2	5 × 20 7 × 30	3.6 5.0	105	32.5	160	344.5	No	0.5
2	F	135	39.4	DORV, D-TGA	Yes	TCPA	1	LSA	2.2	2	5 × 20 7 × 30	2.3 3.2	128	41.1	125	1764.4	No	15
3	F	48	16.0	LVH, D-TGA	Yes	SCPA	1	LSA	1.7	1	5 × 20	2.9	92	22.8	80	368.0	No	96
4	F	20	9.3	DORV, D-TGA	Yes	SCPA	1	LIMA	1.5	2	4 × 10 8 × 30	2.7 5.3	60	10.0	135	225.0	No	71
5	F	41	12.0	TA	Yes	SCPA	1	RSA	1.5	1	5 × 15	3.3	102	29.0	50	259.1	No	38
6	M	57	17.0	HLHS	Yes	TCPA	2	RIMA	1.0	2	3 × 8 4 × 8	3.0 4.0	92	18.4	79	1959.3	No	90
								LSA	2.1	1	7 × 30	3.3						
7	M	162	53.3	DORV, D-TGA	Yes	Shunt *	2	RSA	2.7	4	5 × 20 (*n* = 2) 6 × 20 7 × 30	1.9 2.2 2.6	101	35.0	70	7947.3	No	88
								LSA	3.0	1	5 × 20	1.7						
8	M	16	11.3	L-TGA, VSD	No	Shunt *	1	Ao	2.3	4	6 × 20 (*n* = 3) 7 × 30	2.6 3.0	106	31.5	70	699.9	No	77
9	M	23	12.0	L-TGA, VSD	No	Shunt *	1	RSA	1.7	3	4 × 20 5 × 20 (*n* = 2)	2.4 2.9	105	36.5	64	909.4	No	70
10	M	77	19.2	L-TGA, VSD	No	Shunt *	3	LSA	1.6	3	5 × 20 6 × 20 7 × 30	3.1 3.8 4.4	102	23.5	55	897.1	Yes ^‡^	16
								LIMA	2.7	3	7 × 30 8 × 30 (*n* = 2)	2.6 3.0						
								LIMA	2.3	2	5 × 20 6 × 20	2.2 2.6						
11	F	4	5.7	RVH, PS, VSD	Yes	None	1	Ao	1.8	2	4 × 10 5 × 20	2.2 2.8	68	15.7	27	92.5	No	42
12	F	26	12.2	TA	Yes	SCPA	1	RSA	1.9	4	4 × 8 4 × 10 5 × 15 5 × 20	2.1 2.1 2.6 2.6	123	26.7	20	262.2	No	84
13	F	35	14.1	TA	Yes	SCPA	1	LIMA	1.9	2	4 × 15 5 × 15	2.1 2.6	122	13.2	55	238.9	No	69
14	M	118	24.7	TA	Yes	TCPA	1	Ao	1.7	4	4 × 10 (*n* = 2) 5 × 10 (*n* = 2)	2.4 2.9	73	20.5	70	1142.5	No	71
15	M	12	8.8	D-TGA	No	ASO	1	RSA	1.7	2	4 × 10 5 × 15	2.4 2.9	84	22.3	40	275.4	No	47
16	M	52	12.5	LVH, AoH	Yes	SCPA	2	Ao	1.8	2	4 × 10 5 × 15	2.2 2.8	163	23.8	42	364.1	No	38
								RSA	2.0	2	4 × 8 5 × 15	2.0 2.5						
17	F	39	12.0	DORV, LVH	Yes	SCPA	2	LSA	1.5	3	3 × 8 5 × 15 5 × 20	2.0 3.3 3.3	132	35.0	54	295.3	No	26
								RIMA	2.4	3	7 × 30 8 × 30 (*n* = 2)	2.9 3.3						
18	F	46	13.0	DORV, LVH	Yes	SCPA	1	Ao	1.8	1	4 × 10	2.2	85	16.2	60	139.4	No	19
19	M	117	32.7	TA	Yes	TCPA	2	LSA	2.3	4	4 × 8 4 × 10 6 × 20 (*n* = 2)	1.7 1.7 2.6	104	22.3	155	3106.4	No	78
								LIMA	2.1	2	5 × 15 6 × 20	2.4 2.9						
20	M	7	8.1	PDA	No	none	2	Ao	1.6	1	4 × 10	2.5	66	11.8	30	102.4	No	64
								Ao	2.0	2	4 × 8 5 × 15	2.0 2.5						
21	F	10	7.0	DILV, D-TGA	Yes	Shunt *	2	LIMA	2.0	3	5 × 15 6 × 20 (*n* = 2)	2.5 3.0	75	25.5	17	108.3	No	95
								LSA	2.5	1	4 × 15	1.6						
22	F	42	14.9	DILV, D-TGA	Yes	SCPA	2	RSA	1.6	3	4 × 10 5 × 15 5 × 20	2.5 3.1 3.1	90	25.5	12	455.0	No	63
								RIMA	2.4	1	7 × 30	2.9						
23	M	53	13.0	DILV, PA	Yes	TCPA	2	RIMA	2.5	2	5 × 20 (*n* = 2)	2.0	111	28.1	132	588.0	No	77
								LBV	3.1	3	4 × 10 7 × 30 8 × 30	1.3 2.3 2.6						
24	M	81	19.6	TA	Yes	TCPA	1	RIMA	1.5	1	5 × 20	3.3	71	9.7	64	268.6	No	43
25	M	168	45.0	AVSD, LVH	Yes	TCPA	1	RBV	2.6	4	7 × 30 (*n* = 2) 8 × 30 (*n* = 2)	2.7 3.1	76	16.1	65	283.2	No	60
26	M	14	10.9	D-TGA	No	ASO	1	RSA	2.0	3	4 × 10 (*n* = 2) 5 × 15	2.0 2.5	49	17.3	75	657.5	No	71
27	F	49	14.8	PA, VSD	No	Shunt *	1	Ao	3.7	3	7 × 30 (*n* = 2) 8 × 30	1.9 2.2	133	46.0	110	773.4	No	93
28	M	40	11.0	DORV, LVH	Yes	SCPA	1	RSA	2.1	2	5 × 15 5 × 20	2.4 2.4	122	35.5	34	435.4	No	38
29	M	42	13.2	DORV, LVH	Yes	TCPA	1	RCA	2.4	2	4 × 10 5 × 15	1.7 2.1	80	16.8	65	413.6	No	36
30	M	490	64.0	PA, VSD	Yes	none	2	RSA	2.4	3	7 × 30 8 × 30 (*n* = 2)	2.9 3.3 2.4	79	20.7	123	6955.2	No	36
								LSA	2.5	2	6 × 20 7 × 30	2.8						
31	M	206	59.0	TA	Yes	TCPA	1	LIMA	1.6	3	5 × 15 (*n* = 2) 5 × 20	3.1 3.1	82	20.0	115	3094.8	No	63
32	M	71	18.5	HLHS	Yes	TCPA	1	Ao	1.4	1	3 × 8	2.1	84	19.9	85	706.2	No	23
33	F	31	10.0	TAC	No	TAC repair	1	RIMA	2.1	3	6 × 20 (*n* = 2) 7 × 30	2.9 3.3	51	18.4	90	713.0	No	19
34	M	155	39.2	DORV, D-TGA, LVH	Yes	TCPA	1	LIMA	1.3	1	4 × 10	3.1	82	19.7	100	1438.3	No	34
35	M	9	7.8	DILV, TGA, PS	Yes	SPCA	1	RSA	1.8	2	4 × 8 4 × 10	2.2 2.2	52	19.2	18	59.0	No	34
36	M	20	12.3	DORV, TGA, LVH	Yes	SPCA	2	Ao	1.6	1	4 × 10	2.5	101	25.9	39	442.6	No	30
								RIMA	1.5	2	5 × 15 5 × 20	3.3 3.3						
37	F	8	7.0	TA	Yes	Shunt *	2	Ao	1.5	3	3 × 4 3 × 8 (*n* = 2)	2.0 2.0	82	20.0	15	61.2	No	91
								RSA	1.8	1	4 × 10	2.2						
38	F	24	9.6	TA	Yes	SPCA	1	LSA	1.5	1	5 × 15	3.3	66	11.0	126	204.8	No	75
39	M	232	50.0	TA	Yes	TCPA	1	LBV	2.9	3	4 × 10 6 × 20 7 × 30	1.4 2.1 2.4	52	11.7	95	3785.0	No	95
40	F	183	44.0	DILV, D-TGA	Yes	TCPA	1	LCCA	2.0	1	5 × 20	2.5	131	34.2	60	5752.8	No	75
41	F	219	50.3	DILV, D-TGA	Yes	TCPA	1	RSA	1.8	2	3 × 8 5 × 15	1.6 2.8	110	29.5	65	5157.2	No	39
42	M	2	4.2	D-TGA, VSD, PS	Yes	Shunt *	1	Ao	2.3	2	5 × 20 (*n* = 2)	2.2	49	9.5	38	61.7	No	60
43	M	148	37.9	TA	Yes	TCPA	1	SVC	3.0	3	7 × 30 (*n* = 2) 8 × 30	2.3 2.7	75	16.5	121	860.7	No	64
44	M	28	10.6	DORV, LVH	Yes	SPCA	1	LSA	2.8	2	5 × 15 5 × 20	1.8 1.8	117	32.5	20	231.6	No	66
45	M	75	16.2	DORV, LVH	Yes	TCPA	1	LBV	2.9	3	5 × 20 7 × 30 (*n* = 2)	1.7 2.4	60	12.9	12	200.0	No	19
46	M	8	6.4	DORV, LVH	Yes	SCPA	2	LIMA	1.7	1	5 × 20	2.9	101	27.1	40	102.6	No	86
								RIMA	2.7	3	7 × 30 (*n* = 2) 8 × 30	2.6 3.0						
47	F	25	13.3	DORV, PS, LVH	Yes	SCPA	1	RIMA	1.7	1	4 × 10	2.4	88	17.4	57	292.6	No	48
48	F	36	14.7	DORV, PS, LVH	Yes	SCPA	1	RIMA	1.8	1	4 × 10	2.2	59	15.8	45	233.6	No	37
49	M	37	18.9	TA	Yes	SCPA	1	LIMA	1.9	3	4 × 10 5 × 15 (*n* = 2)	2.1 2.6	73	17.4	100	701.0	No	69

Ao = aorta; AoH = aortic hypoplasia; ASO = arterial switch operation; AVSD = atrio–ventricular septal defect; DILV = double inlet left ventricle; DORV = double outlet right ventricle; D-TGA = dextro-transposition of the great arteries; HLHS = hypoplastic left heart syndrome; LBV = left brachiocephalic vein; LCCA = left common carotid artery; LIMA = left internal mammary artery; LSA = left subclavian artery; LVH = left ventricular hypoplasia; L-TGA = levo-transposition of the great arteries; PA = pulmonary atresia; PDA = patent ductus arteriosus; PS = pulmonary stenosis; RBV = right brachiocephalic vein; RCA = right coronary artery; RIMA = right internal mammary artery; RSA = right subclavian artery; RVH = right ventricular hypoplasia; SCPA = superior cavo–pulmonary anastomosis; SPC = Systemic-to-pulmonary collateral(s); SVC = superior vena cava; TA = tricuspid atresia; TAC = truncus arteriosus communis; TCPA = total cavo–pulmonary anastomosis; TOF = tetralogy of Fallot; VSD = ventricular septal defect; ^‡^ vascular injury as described in the main text; * Blalock-Taussig shunt or central aorto–pulmonary shunt.

## Data Availability

The data underlying the present study are available on request (corresponding author).

## Abbreviations

CHD	congenital heart disease
CHM	Concerto™ Helix microcoil (nylon-fibered)
MAPCA	major aorto–pulmonary collateral arteries
SCPA	superior cavo–pulmonary anastomosis
SPC	systemic-to-pulmonary collateral(s)
TCPA	total cavo–pulmonary anastomosis
